# Ensuring access to justice: the need for community paralegals to end AIDS by 2030

**DOI:** 10.1002/jia2.26146

**Published:** 2023-08-03

**Authors:** Kati Hinman, Nina Sun, Joseph J. Amon

**Affiliations:** ^1^ Office of Global Health Dornsife School of Public Health Drexel University Philadelphia Pennsylvania USA

**Keywords:** HIV, prevention, access to care, structural interventions, human rights, legal services

## Abstract

**Introduction:**

The HIV response has long recognized that certain “key populations” such as individuals in detention, adolescent girls and young women, sex workers, people who use drugs, LGBTQ individuals, migrants and others face higher barriers to access to, uptake of, and retention in HIV prevention and treatment services. One approach to addressing these barriers is the training of community paralegals to advocate for the rights of individuals and to address discrimination in health settings.

**Discussion:**

Community paralegal programmes have been able to successfully address rights violations that impact access to health services and underlying determinants of health across a range of countries and populations, focusing upon issues such as discrimination and the denial of health services; unlawful detention of outreach workers, sex workers, persons who use drugs and men who have sex with men; and harmful traditional practices and gender‐based violence. In addition to resolving specific cases, evaluations of paralegal programmes have found that these programmes increased legal literacy among key populations at risk of HIV and increased understanding of human rights among healthcare providers, resulting in improved access to HIV services. Some evaluations have noted challenges related to the sustainability of paralegal programmes similar to those raised with community health worker programmes more broadly.

**Conclusions:**

To achieve global HIV goals, funding for legal literacy and paralegal programmes should be increased and interventions should be rigorously evaluated. Efforts should target discrimination in access to HIV prevention and treatment and criminalization of key populations, two key barriers to ensuring access to HIV prevention and treatment services.

## 1 INTRODUCTION

The Global AIDS Strategy (2021–2026) sets out a roadmap with priority actions to help countries and communities achieve an end of AIDS as a public health threat by 2030. Specifically, the Strategy outlines the need to break down barriers to achieving HIV outcomes, including ensuring access to justice as a critical component to creating a supportive legal environment, and eliminating the harmful impact of criminalization, police abuse and human rights violations on vulnerability to infection and access to care [1]. Ongoing discrimination against key populations—including sex workers, transgender people, gay and bisexual men and other men who have sex with men, people who inject drugs and prisoners—as well as people living with HIV (PLH) more generally, has contributed to the increasing incidence in these populations even in places where overall HIV incidence is decreasing [2]. According to a 2022 UNAIDS report, 70% of new HIV infections were among key populations and their sexual partners, despite these groups representing less than 5% of the global population [3]. Punitive laws and discriminatory practices that target members of key populations not only threaten their lives and dignity, but also increase their vulnerability to HIV infection and limit access to treatment [2, 4, 5].

One strategy for addressing legal barriers and human rights violations that impede access to HIV prevention and treatment is the use of paralegal professionals and trained community (or “peer”) paralegals. Community paralegal programmes, which identify and train individuals to work with their own communities, often concentrate on educating and empowering individuals to know their rights and to be able to seek accountability for abuses, depending upon their level of training and via formal judicial systems or alternatives forms of mediation [6, 7] (Table 1). Using community paralegals to play this role facilitates broader access to justice generally, reinforcing, for example, non‐discrimination and access to HIV services for all.

**Table 1:** Illustrative types of legal aid services provided by paralegals by level of training

Level of training	Types of legal aid services provided by paralegals
Basic	Basic legal education Legal information Orientation and referral Accompaniment of clients to mediation or legal proceedings Mediation on less complicated matters
Intermediate	Legal education Legal information Legal advice Orientation, referral, accompaniment and mediation Alternative dispute resolution
Comprehensive	Full spectrum of primary legal aid services (as described above) Legal assistance

*Source*: Adapted from: Access to Justice. Global Justice Project. Zambia National Report [13].

The idea of engaging paralegals in the HIV response is not new. Lawyers and paralegals have worked with PLH from the start of the epidemic [8, 9]. In sub‐Saharan Africa, community paralegals were involved in efforts to secure widows’ and orphans’ rights, ensuring widow inheritance, helping widows write wills and secure ownership of their land and homes, and protecting children from exploitation [10].

Yet historically, community paralegal programmes, especially those linked to health services, have operated at a small scale, with limited funding and inadequate evaluation. In 2012, UNAIDS identified the expansion of HIV‐related legal services and human rights and legal literacy programmes as “key programmes” needed to overcome barriers to “effective national responses to HIV” and “critical enablers to the success of basic HIV prevention and treatment programmes” [11]. The Global Fund to Fight HIV, Tuberculosis and Malaria's *Breaking Down Barriers* initiative—which funds human rights programmes that support HIV services in 20 countries—has been one of the biggest funders of community paralegal programmes in the HIV response. In 2020–21, a mid‐term assessment of the initiative found that efforts to scale up legal literacy interventions and train community paralegals can create more enabling social and legal environments for the HIV response, as well as reduce HIV‐related vulnerabilities [12]. Building upon these results, we argue that: (1) community paralegal programmes should be expanded; and (2) such programmes should include rigorous evaluation, assessing the effects of paralegals on both access to justice and key health outcomes.

## 2 DISCUSSION

### 2.1 History of community paralegals within HIV and other health services

Among the earliest incarnations of programmes addressing health and justice was the creation of medical−legal partnerships and “street lawyers”—community paralegals who provided legal aid and welfare support to people who use drugs, sex workers and individuals experiencing homelessness [14, 15]. Building on these programmes, the Open Society Foundations supported HIV‐related legal empowerment programmes over the course of two decades, including the training of paralegals in Eastern Europe/Central Asia and sub‐Saharan Africa [16]. In these programmes, the use of community paralegals emerged as a core activity to support members of key and vulnerable populations to access HIV prevention and treatment services.

Though it is difficult to systematically measure the effectiveness of community paralegals on health outcomes, programme assessment and qualitative research have found positive impacts of paralegal programmes and legal literacy campaigns [6]. For example, paralegals in Indonesia and Kyrgyzstan raised awareness of health‐related rights and ensured access to HIV treatment and health services for persons in detention [17, 18]. In Mozambique, the non‐profit Namati trained community paralegals to address barriers to quality healthcare, advocating for patients facing medicine stockouts, facility infrastructure issues (e.g. broken bathrooms, lack of security for patients) and provider performance issues (e.g. breaches in confidentiality, requests for bribes, mistreatment of women in maternity wards) [19]. In Macedonia, Romania and Serbia, clients of paralegals reported increased awareness of their rights, as well as an increased willingness to challenge rights violations. In turn, clients described a decrease in the denial of care, harassment and other abuses by healthcare providers [20]. In Kenya, an assessment of a property rights programme for women, which included paralegal support, reported decreased HIV vulnerability [21]. Additional evaluations of HIV‐related paralegal programmes have found positive results in projects that have worked with men who have sex with men [22]; sex workers [23, 24, 25, 26]; persons who use drugs [27]; prisoners [17]; pregnant women [28]; and widows [17]. These findings illustrate how community paralegal programmes can be a key component supporting effective health systems and addressing health risk (Table 2).

**Table 2:** Range of HIV paralegal programmes, barriers, strategies and outcomes [17, 22, 23, 24, 25, 26, 27, 28]

Populations	Barriers addressed	Paralegal strategies	Outcomes/impact
Adolescent girls and young women Men who have sex with men Transgender women Sex workers Persons who use drugs Prisoners Migrants Ethnic/racial/linguistic minorities Persons with disabilities People living with HIV Pregnant women Widows Survivors of gender‐based violence	Stigma Discrimination Extortion Denial of care Inheritance Police abuse/harassment Access to prevention Access to treatment	Advocacy Mediation Litigation Policy change Enforcement Law reform	Change in social norms
			Increase in: Access Uptake Retention of HIV prevention and treatment

However, despite these findings and despite widespread and long‐term use of paralegal and broader legal empowerment programmes to address barriers to prevention and treatment, rigorous evaluation of the impact of such programmes on specific health outcomes remains limited [16]. This “evaluation gap” is not unique to community paralegal programmes but is noted more generally in relation to peer‐ and community‐led HIV interventions [29, 30].

### 2.2 Training and integrating paralegal professionals into HIV programmes

Recognizing the impact of legal environments on the HIV response, the Global Fund's *Breaking Down Barriers* initiative has supported community paralegal programmes in 17 countries. Several common outcomes were found. First, countries have successfully scaled‐up paralegal trainings to create a cadre of paralegals capable of addressing a range of barriers to HIV prevention and care. Second, within these programmes, there is a growing pattern of integration and/or close linkages of paralegal support with health services. These steps have led not only to the resolution of specific cases, but also increased rights‐awareness among community members and health workers and evidence of improved access to HIV prevention and treatment.

### 2.3 Scaling up community paralegal programmes

Examples of the successful scale‐up of trained paralegals come from a number of countries. In sub‐Saharan Africa, programmes in Senegal, Uganda and Kenya illustrate the range of interventions supported.

In Senegal, since 2017, a total of 118 sex workers from five regions across the country have been trained as paralegals. The trainings provided sex workers general legal information (e.g. on sex work laws and regulations, sexual violence, civil status etc.) as well as specific information on how to document rights violations and file a complaint with the police and when to refer sex workers with more complex claims to legal clinics. Training participants reported an increased understanding of sex workers’ rights and recommended that trainings be expanded [31].

Similarly, in Uganda, the Human Rights Awareness and Promotion Forum (HRAPF) conducted paralegal trainings for a range of key populations, providing legal information, advice, referrals, dispute resolution and representation on cases related to HIV and tuberculosis. With Global Fund support, HRAPF was able to provide stipends to over 80 paralegals across Uganda. In 2020 alone, HRAPF‐funded paralegals handled over 1000 cases related to access to health services [32].

In Kenya, paralegals form part of crisis management teams which include an advocacy officer and an outreach worker. Since 2017, the National AIDS and STI Control Program has trained more than 395 community paralegals on HIV and human rights. These crisis management teams are active in all 47 counties across Kenya, responding to human rights violations against key populations, including 7000 cases of violence against female sex workers in 2020 [33]. Key population members reporting sexual violence were then linked to HIV testing services, facilitating access to post‐exposure prophylaxis [27]. Recognition of the value of paralegals in Kenya is reflected in the 2016 Legal Aid Act, which permits accredited paralegals to provide advice that was formerly in the exclusive purview of lawyers [17, 33].

### 2.4 Integrating paralegal support in healthcare services

The integration of paralegal interventions with health services ensures that such programmes are not seen as antagonistic by healthcare providers but rather as seeking to “problem solve” and ensure universal access to care. This is illustrated in Botswana, where Sisonke Botswana and BONELA's “Improving Pathways to Justice for Sex Workers” programme combines referrals to sexual and reproductive health services, HIV and sexually transmitted infections screening, and HIV treatment with legal literacy workshops and community paralegal outreach. Programme evaluations have found that the programme has resulted in sex workers who are not only more “rights literate,” but better equipped to insist on police investigations and the prosecution of cases, leading to convictions and jail sentences for violent perpetrators [34].

**Figure 1:**
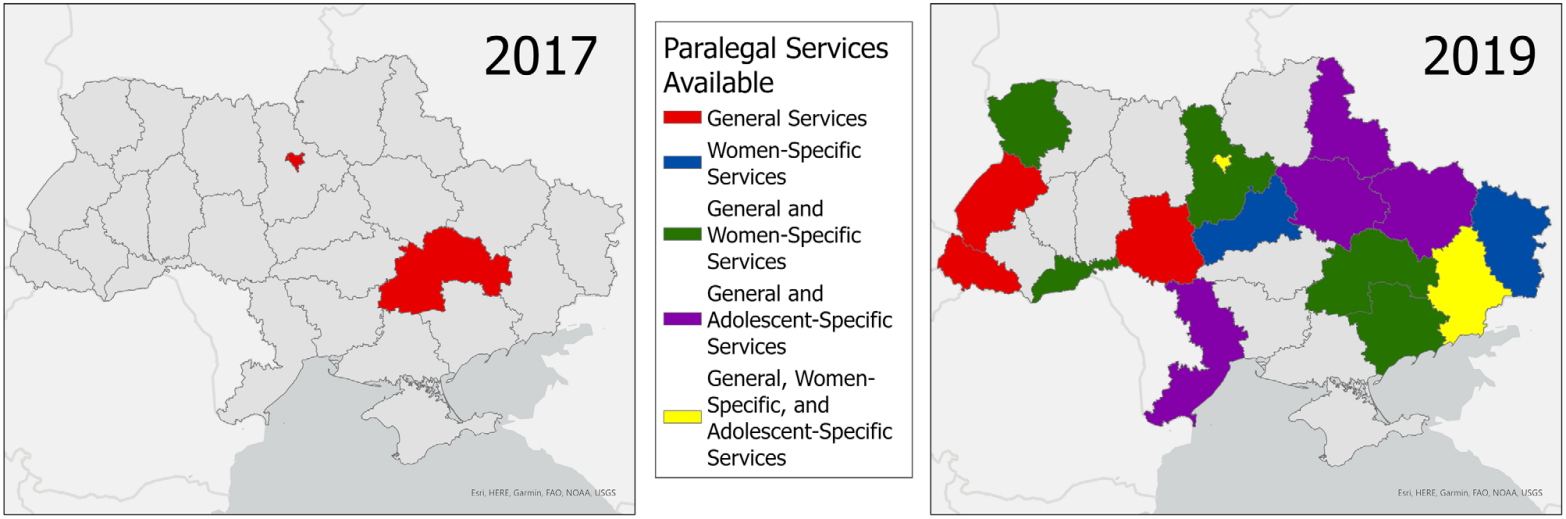
Change in the availability of paralegal support for people who use drugs in Ukraine [36].

In Indonesia and Ukraine, paralegal professionals have been integrated with treatment and prevention interventions, respectively. In Indonesia, the 4 Pillars Program, operated by the Indonesia AIDS Coalition, has implemented paralegal support programmes in 23 districts, using a team that includes a paralegal, a paralegal assistant and a treatment access specialist to reach PLH unlinked to care [35]. In Ukraine, paralegal services for people who use drugs have been scaled up and closely linked with HIV prevention services (Figure 1). The paralegal services, provided by Volna and Vona work closely with harm reduction providers, while paralegals supported by Legalife, a sex worker community organization, provide legal information and referrals to legal services via community outreach and through a national telephone hotline, in addition to also giving out health information, condoms and lubricants, and referrals to sex worker‐friendly doctors [36].

### 2.5 Positive impacts of community paralegals on access to HIV prevention and treatment

Increasing the number of trained paralegals, and the collaboration between paralegals and health professionals, are important first steps towards the ultimate goals of HIV‐related paralegal programmes: to increase access, uptake and retention in HIV prevention and treatment services for key and other vulnerable populations. Increasingly, evidence shows the impact of these programmes at both the individual and population level. In Mozambique, for example, community paralegals helped secure the release from detention of 45 sex workers in Tete province, as well as the removal of adolescent girls from early marriages [37].

From 2018 to 2020 in Botswana, almost 1000 cases were documented by paralegals on issues such as the denial of access to health services, gender‐based violence, stigma and discrimination, abuses against sex workers, child rights violations and police brutality. In some instances, paralegals were able to effect immediate change. For example, after recording instances where clients were unable to access health services because they lacked identity cards, BONELA met with the Director of Immigration and Nationality to raise this concern. After this meeting, the Director assigned staff to address this barrier, facilitating the issuance of new identity cards and access to HIV care [34].

Peer paralegal programmes have also increased legal literacy, empowering communities to raise attention to, and resolve, barriers to HIV prevention and treatment. For example, the Ghana‐West Africa Program to Combat AIDS and STI (WAPCAS) reported in 2020 that 148 cases involving gender‐based violence against sex workers, and extortion, blackmail or threats of extortion against men who have sex with men were brought forward by paralegals working with key populations. Though some cases were referred to lawyers, most cases were resolved by community paralegals, protecting individuals from harm and HIV‐related risks [38]. An evaluation found that awareness among key populations of the existence of the programme was high and positive [39].

Similarly, in Indonesia, a 2020 evaluation of a community paralegal programme found increased awareness of human rights among key populations and community members, local officials and the media. The evaluation also found the increased capacity of civil society organizations and key populations to advocate both locally and nationally for their rights, as well as improved relations with healthcare facilities and healthcare workers, leading to increased access to care. The programme was viewed as strongly positioned for sustainability due to its integration with the health system [35].

### 2.6 Challenges

Despite promising results from paralegal programmes in increasing access to justice, improving enabling environments for service access and facilitating access to HIV prevention and care, significant challenges in terms of funding, sustainability and integrating paralegal programmes into health systems remain. First, across several countries, stakeholders have expressed concern about the low‐paying, often voluntary nature of community paralegal work. Paralegal responsibilities are often on top of other obligations, and limited resources for community paralegals have resulted in high turnover in some programmes. While recognizing the importance of providing legal aid and expanding community paralegal programmes, donors provide limited financial support and with limited exceptions (e.g. Kenya), community paralegals are rarely formally recognized under the legal aid system. While paralegals can be effective and useful in all legal environments, formal recognition of paralegals indicates that governments may be more willing to provide domestic funding for this type of assistance.

Secondly, although integrating paralegals into health systems or interdisciplinary outreach teams is most likely to ensure expanded access, uptake and retention in HIV prevention and treatment for key populations, traditional stakeholders may resist such efforts. Including specific indicators for paralegal work—for example, by tracking output on the number of cases resolved and measuring the number of cases linked to HIV services—could help demonstrate the synergistic impact of integrating paralegals while improving evidence of quality, impact and cost‐effectiveness. Resistance may also come from the recognition of the difficulty of achieving impact in the context of weak judicial systems. Community paralegals, however, can overcome this obstacle by focusing on redress within communities—through apologies, negotiations and promises of non‐repetition—rather than through formal court systems.

Despite considerable evidence of the positive impact of community paralegal programmes, there remains limited evidence from rigorous evaluation studies focused on access, uptake and retention within HIV prevention and treatment services and to vulnerability to HIV infection more generally. Building on existing qualitative evidence, donors should prioritize further evaluation efforts, measuring HIV and access to justice outcomes and experiences of discrimination, which directly impact an individual's overall health and wellbeing. Donors should also require paralegal programmes to develop robust routine monitoring and evaluation systems to establish feedback and learning mechanisms throughout the life of the programming.

## 3 CONCLUSIONS

Community paralegal programmes can be seen as structural interventions that address barriers to HIV services across all socio‐ecological levels [40]. As such, they act on the social, economic and political factors that influence individual, community and societal health outcomes [41]. Yet, despite decades of evidence of the importance of empowered communities claiming their rights [29, 42], structural interventions designed to reduce new HIV infections, increase access to HIV prevention, and improve uptake and retention in HIV treatment remain under‐studied and under‐implemented, and many key populations and people living with HIV lack understanding of their right to health and non‐discrimination outlined in domestic and international law [43].

Expanded investment and support of community paralegals as part of HIV and other health services is essential in reducing structural inequities and advancing the right to health. This applies both in the context of reducing barriers to access healthcare, as well as in addressing the underlying determinants of health, especially for populations experiencing marginalization and criminalization. Community paralegal programmes address the specific gap that HIV leaders have identified as critical to achieving an “end” of AIDS: reaching those “left behind” and reaching 90‐90‐90 goals [44].

It is important with an increased investment to also see an increased commitment to evaluating the impact of paralegal programmes. Increased evidence of effectiveness from research on diverse approaches to the provision of paralegal services will also help to address sustainability challenges by building greater commitment from donors and governments. Measuring the impact of structural interventions such as paralegal interventions is less straightforward than assessing biomedical interventions or behaviour change but is no less important. As with research to measure the impact of structural racism [45], the evaluation of structural interventions to reduce health disparities [46] and overcome human rights barriers to HIV services [47] requires methodological innovation, including mixed‐methods and multi‐level models to assess impact. In spite of the widespread acceptance of randomized controlled trials (RCTs) as the “gold standard” for evaluating interventions, RCTs are often limited in their explanatory power and, in the case of structural interventions tailored to specific environments, in their generalizability. This is particularly true for the evaluation of “complex” interventions, such as paralegal interventions, which address multiple barriers to HIV services and affect multiple HIV outcomes (including HIV vulnerability, access to prevention, uptake and retention in care).

HIV‐focused community paralegal programmes can also support health systems beyond HIV, as evidenced by the response of many paralegal programmes to the COVID‐19 pandemic [48]. Whether grounded in communities or linked to health systems, community paralegals and similar legal empowerment approaches provide a mechanism of accountability and a means to counter institutionalized discrimination, cutting across disease‐specific aims, and strengthening civil society engagement and the rule of law. In conclusion, to accelerate progress towards ending AIDS as a public health threat, legal empowerment interventions, like community paralegals, should receive increased funding and institutional support.

## COMPETING INTERESTS

The authors report no competing interests.

## AUTHORS’ CONTRIBUTIONS

JJA conceptualized the article. KH, NS and JJA contributed to the development of the first draft and to subsequent revisions.

## FUNDING

This article was written without specific donor funding.

## Data Availability

Data sharing is not applicable to this article as no datasets were generated or analysed during the current study.
